# Changes in Tissue Composition and Load Response After Transtibial Amputation Indicate Biomechanical Adaptation

**DOI:** 10.1007/s10439-021-02858-0

**Published:** 2021-09-27

**Authors:** J. L. Bramley, P. R. Worsley, D. L. Bader, C. Everitt, A. Darekar, L. King, A. S. Dickinson

**Affiliations:** 1 School of Engineering, Faculty of Engineering and Physical Sciences, University of Southampton, Mailpoint M7, University Road, Southampton, SO17 1BJ UK; 2 School of Health Sciences, Faculty of Environmental and Life Sciences, University of Southampton, Southampton, UK; 3 University Hospital Southampton NHS Foundation Trust, Southampton, UK

**Keywords:** Transtibial amputation, Magnetic resonance imaging, Infiltrating adipose, Remodelling, Muscle atrophy

## Abstract

Despite the potential for biomechanical conditioning with prosthetic use, the soft tissues of residual limbs following lower-limb amputation are vulnerable to damage. Imaging studies revealing morphological changes in these soft tissues have not distinguished between superficial and intramuscular adipose distribution, despite the recognition that intramuscular fat levels indicate reduced tolerance to mechanical loading. Furthermore, it is unclear how these changes may alter tissue tone and stiffness, which are key features in prosthetic socket design. This study was designed to compare the morphology and biomechanical response of limb tissues to mechanical loading in individuals with and without transtibial amputation, using magnetic resonance imaging in combination with tissue structural stiffness. The results revealed higher adipose infiltrating muscle in residual limbs than in intact limbs (residual: median 2.5% (range 0.2–8.9%); contralateral: 1.7% (0.1–5.1%); control: 0.9% (0.4–1.3%)), indicating muscle atrophy and adaptation post-amputation. The intramuscular adipose content correlated negatively with daily socket use, although there was no association with time post-amputation. Residual limbs were significantly stiffer than intact limbs at the patellar tendon site, which plays a key role in load transfer across the limb-prosthesis interface. The tissue changes following amputation have relevance in the clinical understanding of prosthetic socket design variables and soft tissue damage risk in this vulnerable group.

## Introduction

Following lower limb amputation, the residual skin and soft tissues form a critical interface with the bespoke ‘socket’ component of a prosthetic limb. These tissues are vulnerable to damage, particularly during the early rehabilitation phase, prior to adequate biomechanical conditioning arising from mechanical loading.^35^ The resulting tissue deformations may cause skin and soft tissue damage, with reported prevalence between 36 and 66%.^12,35,36^

Experimental and numerical models indicate that large deformations over short periods of time represent the most important factor in the causal pathway for Deep Tissue Injury (DTI), which initiates in muscle tissues.^8,15,31–33,39,40,53,54^ By contrast, superficial pressure ulcers (PUs) are generally caused by external pressures and shear forces. The tissue tolerance to loading magnitude and duration varies between individuals,^19^ and is influenced by many intrinsic factors.^10^ There has been relatively little research into skin damage in individuals with lower limb amputations, despite the specific risk factors and high prevalence in this group.^18^ Indeed the residual limbs are exposed to challenging biomechanical conditions, impaired load tolerance due to comorbidities, considerable variability in anatomy and surgical reconstruction, and the presence of scar tissue over vulnerable sites.^41^

Tissue loading at the residuum-prosthesis interface is influenced by the socket design, with the prosthetist considering both the morphology of the local tissues and their load tolerance.^28,42,46^ These characteristics change post-amputation due to oedema, muscle atrophy and tissue remodelling in biomechanical adaptation to prosthetic load bearing. The oedematous response to the trauma of amputation decreases gradually in the months following surgery.^29^ Physiotherapy exercises are prescribed to promote range of motion in the residual limb joints, and reduce muscle atrophy and oedema.^43^ Despite these interventions, residual muscles atrophy due to denervation and disuse, with a subsequent infiltration of adipose or fibrous tissues.^51,58^ In addition, the superficial tissues adapt in response to increased repetitive loading, with pressures and shear stresses at the limb-prosthesis interface ranging widely, from 4 to 938 mmHg (0.5 to 125 kPa) and 8 to 389 mmHg (1 to 52 kPa), respectively.^56,62,63^ The skin and subdermal tissues may thicken, and callus is formed to adapt their vascular function. Such changes have been reported using optical coherence tomography, with an increased epidermal thickness in transtibial residua compared to the contralateral limb, and higher microvascular function.^55^

To date there is limited evidence of how biomechanical loading affects the vulnerable residuum muscle and adipose tissues during early rehabilitation. Volume imaging modalities have been used to observe residual limb adaptation. These include Magnetic Resonance Imaging (MRI) to visualise muscle morphology changes and differentiate between changes due to oedema and muscular atrophy,^28^ and computed tomography (CT) to determine the proportion of muscle and fat mass in the residual limb compared to contralateral limbs.^50^ MRI has been used to evaluate fatty infiltration in other tissues,^20,21^ but previous studies have not quantified adipose tissue or distinguished between superficial and intramuscular distribution in amputees. This is despite the recognition that intramuscular fat levels represent an indicator of the risk for severe pressure ulcers, as observed in the gluteal region of individuals with spinal cord injury prone to DTI.^59^ Furthermore, it remains unclear how these changes may alter tissue tone and stiffness, which represent key biomechanical characteristics at the interface with the prosthetic socket, likely to influence residuum-socket load transfer patterns and in turn the soft tissue damage risk. Indeed, these properties have been shown to change due to a range of factors relevant to the amputee population including ageing, stroke, exercise and post-exercise massage,^2,9,14,25^ as well as in the spinal cord injury population.^48^

Changes in both soft tissue morphology and mechanical response to loading following amputation have clinical relevance to the understanding of effective prosthetic socket designs. Research has identified the potential application of prosthesis-limb interface sensors and/or numerical predictions to support the prevention of soft tissue damage.^11,45^ However, there is still limited research which assesses the composition and structural features which are critical in determining tissue tolerance to mechanical loading. Accordingly, this study was designed to characterise residual limb soft tissue morphology, composition and mechanical response to representative prosthetic loading. To assess changes arising from amputation and prosthetic limb use, the study compared residual and intact limbs of individuals with unilateral transtibial amputation, and intact controls. This involved characterising the proportions of both superficial adipose and adipose infiltrating muscle tissue using MRI, their deformation and gross strain under *in situ* mechanical loading with indenters, and measuring the structural stiffness of the combined soft tissue layers.

## Methodology

### Study Design and Recruitment

An observational comparison study was conducted with participants recruited from the local community population, including those with and without unilateral transtibial amputation. Inclusion criteria involved participants over 18 years of age, in good health with no active skin-related conditions at sites relevant to the study. Participants without amputation had additional exclusion criteria of neurological and vascular pathologies. Local Ethics Committee approval for the test protocol was granted by the University of Southampton (ERGO IDs: 29696 and 41864) and participants provided informed consent in writing.

### Test Protocol

**Figure 1:**
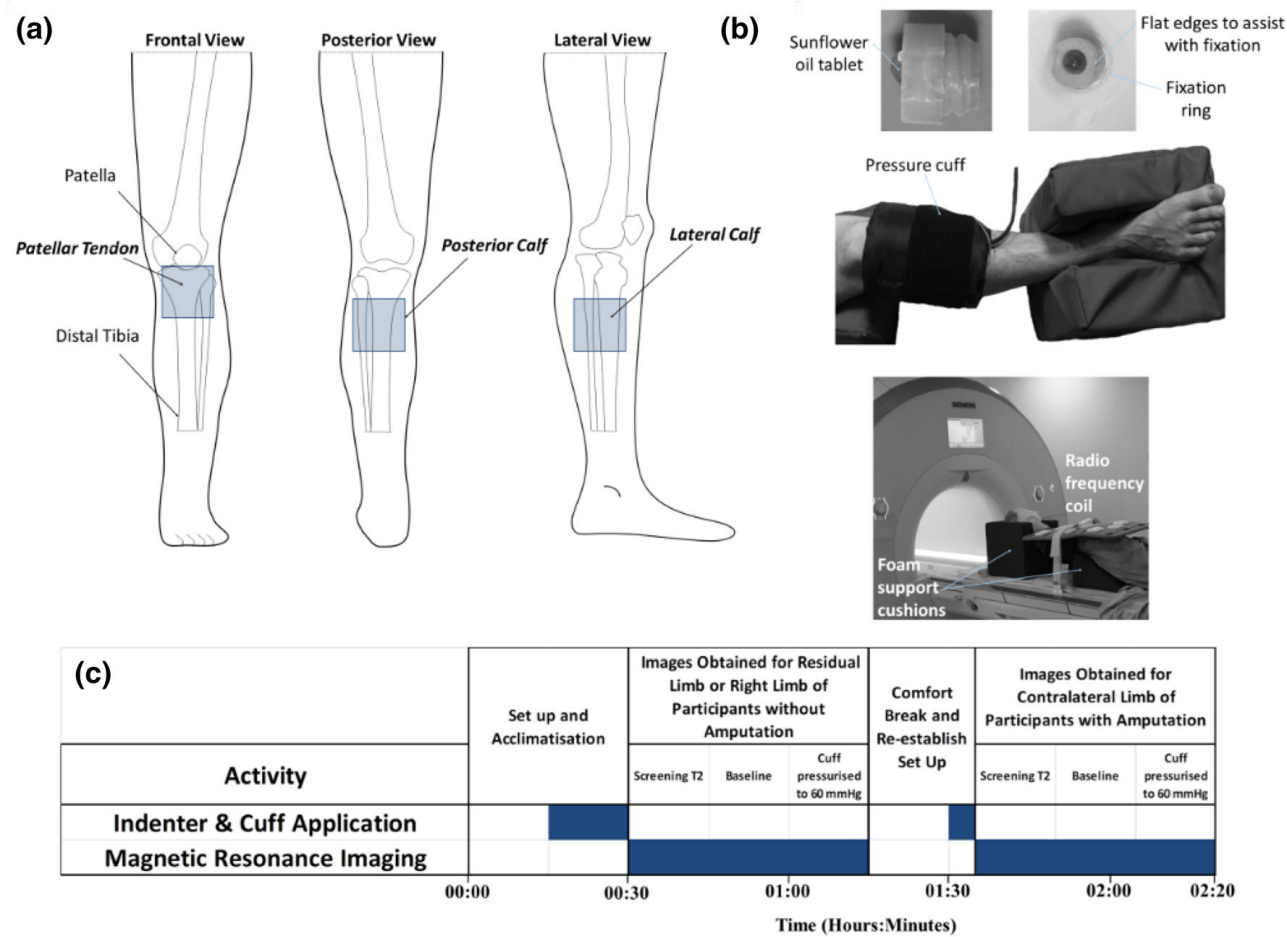
(a) Measurement sites on the right lower limb, each of area 50 × 50 mm; (b) 3D printed indenter positioned at each measurement site *via* adhesive fixation ring, enclosed by a pressure cuff with the limb in the supported test position (middle) and MRI test set up prior to imaging (bottom), (c) Timeline of the MRI test protocol.

Pressure was applied to the right proximal calf of control participants without amputation, and both calves of participants with unilateral transtibial amputation using an inflatable cuff (Ref 0124 Aneroid Sphygmomanometer, Bosch + Sohn GmbH, Germany) according to a previous publication.^6^ A prosthetic liner (6mm ContexGel Liner, NMA21L200/XXL, RSL Steeper, UK) was positioned underneath the cuff to provide a representative material to interface with the skin. Three 50 mm square sites were selected for measurement on each limb representing load bearing regions of differing tissue composition, namely the patellar tendon, lateral calf, and posterior calf (Fig. 1a). Cylindrical polymer indenters of 17 mm diameter and 15 mm height were positioned underneath the cuff at the three measurement sites, containing sunflower oil capsules to facilitate identification within the MR images (Fig. 1b).

Participants were scanned supine, feet-first using a 3T MRI scanner (MAGNETOM Skyra, Siemens, Germany), with their test-limb elevated and resting on foam supports. MR images were acquired using an 18-channel body array coil placed on top of the test limb, as well as the spine coil in the scanner couch underneath the limb. Images were acquired at baseline and at a cuff inflation pressure of 60 mmHg (8 kPa) to characterise direct tissue deformation and visualise morphology and tissue composition^6^ (Fig. 1c). Volumetric MRI data were acquired using a 3D T1 DIXON sequence^34^ with an echo time (TE) of 6.15 ms and a repetition time (TR) of 17.10 ms, a 134 mm field of view, an in-slice resolution of 0.6 mm × 0.6 mm and a corresponding slice thickness of 1.2 mm. The acquisition time was 5 min 19 s. This sequence generates a set of 4 volumetric axial image datasets, each with a different contrast: in-phase, opposed-phase, fat-only and fat-suppressed (water-only) images.

**Figure 2:**
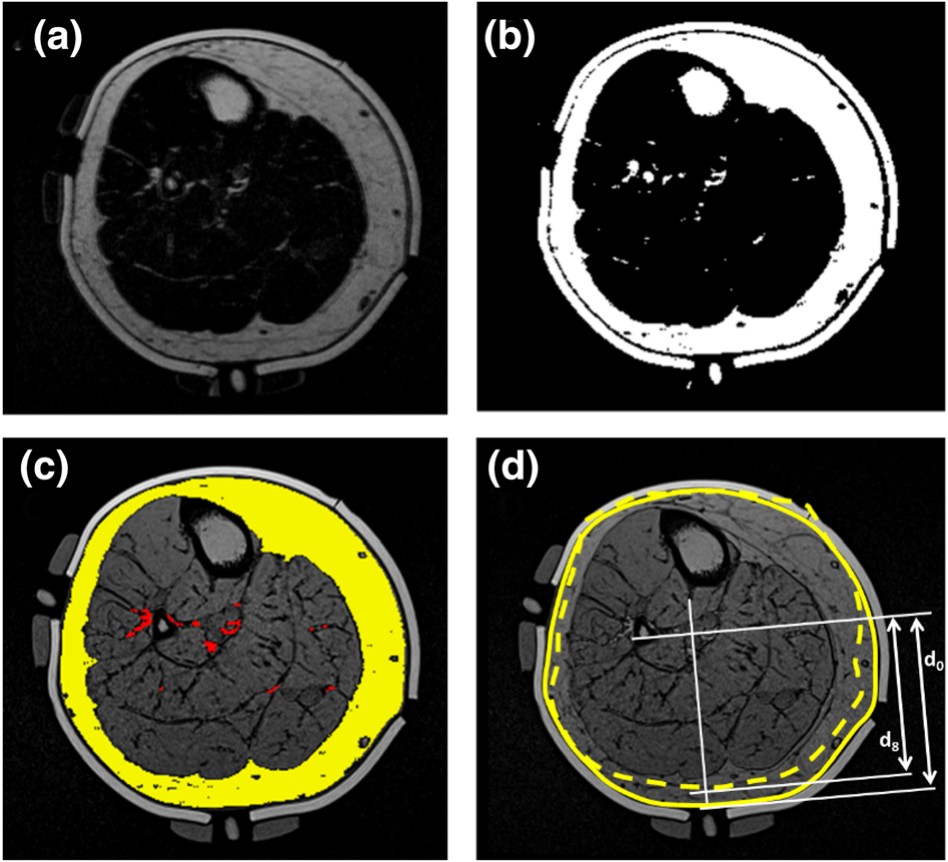
Image processing steps applied to the axial MRI fat-only slice of the lower limb at the posterior calf measurement site, showing (a) original image, and (b) after binarization and masking. (c) superficial adipose mask (yellow) and muscle-infiltrating adipose mask (red) superimposed over the corresponding opposed-phase image at same slice, and (d) superimposed outlines of limb under uninflated cuff baseline (solid line) and 8 kPa inflated cuff (dashed line) conditions. Example measures are shown for calculating the displacement and gross strain arising from cuff inflation between the posterior calf indenter to the nearest bony prominence, uninflated (*d*_*0*_) and at 8 kPa inflation (*d*_*8*_).

The volume of both superficial- and muscle-infiltrating adipose tissue was quantified by processing the MR images in ImageJ 1.52p (Rasband, W. National Institute of Health, US). Background noise was removed by subtracting a pixel intensity of 10, and binary images created with the Auto Threshold Stack tool. Masks were created representing the whole soft tissue area, tibia, fibula and muscle, and Boolean operations were applied to generate superficial- and muscle-infiltrating adipose tissue masks whose areas were calculated (Fig. 2). Gross deformation and compressive strain under each indenter was estimated by selecting single MR slices corresponding to the centre of the measurement sites, measuring the normal distance from the indenter surface to the nearest bony prominence and comparing these values at both unloaded baseline (0 kPa) and inflated cuff test conditions (8 kPa). Deformation was calculated as $\delta = \left({d}_{0}-{d}_{8}\right)$ and strain as $\varepsilon = \left({d}_{0}-{d}_{8}\right)/{d}_{0}$.

Prior to imaging, interface pressure and soft tissue stiffness measurements were recorded for each participant in a seated position on a commercial hospital bed with adjustable backrest (Enterprise, Arjo Huntleigh, Bedforshire, UK), with their test-limb elevated and resting on foam supports. Indenter-skin interface pressures were measured using a pneumatic pressure monitoring system (Mk III, Talley Medical, Romsey, UK) with 28 mm diameter measurement cells, which have a reported mean error of 12 ± 1% and a repeatability of ±0.53 mmHg.^4^ The cuff and liner were then doffed, and the MyotonPro (Myoton AS, Talinn, Estonia) device was used to apply a 15 ms, 0.4 N mechanical impulse at each of the measurement sites in order to estimate the superficial tissue structural stiffness. This device estimates dynamic stiffness using $S = {a}_{max}\times {m}_{probe}/\Delta l$, where ${a}_{max}$ is the impulse probe’s maximum acceleration, ${m}_{probe}$ is its mass and $\Delta l$ is its displacement at the point of maximum acceleration. The MyotonPRO has been demonstrated to show reliable lower limb skeletal muscle structural stiffness measurements^3^ which correlate with shear wave elastography.^23^

### Data Analysis

Raw data from each of the measurement techniques were processed and analysed using MATLAB (MathWorks, USA) and SPSS Statistics (IBM, USA). After testing for normality, MRI data were analysed using non-parametric descriptors (median, quartiles and range), whereas interface pressure and tissue stiffness data were analysed using parametric descriptors (mean and standard deviation). Differences in tissue composition, deformation and strain between control, contralateral and residual limb groups (non-parametric) were assessed for statistical significance using a Mann-Whitney-U test, and differences between structural stiffness (parametric) were assessed using T-Tests. Relationships between percentage of infiltrating and superficial adipose tissue, the time since amputation, socket use, structural stiffness and deformation were evaluated using scatter plots and Spearman’s correlation. Differences were considered to be statistically significant at the 5% level (*p* < 0.05).

## Results

Ten participants without amputation and 10 participants with unilateral transtibial amputation were recruited (Table 1). The control group was younger than the group with amputation and presented with a lower median weight and BMI. There was a variety of causes of amputation in the latter group, and a wide range of time since amputation, from 1 to 35 years.

**Table 1:** Participant characteristics, reported as median (range).

Characteristic		Controls			Participants with Amputation			
		All (*n* = 10)	Male (*n* = 6)	Female (*n* = 4)	All (*n* = 10)		Male (*n* = 8)	Female (*n* = 2)
Age (years)		28 (23–36)	26 (23–34)	28 (27–36)	41 (25–62)		45 (25–62)	38 (30–46)
Height (m)		1.78 (1.60–1.92)	1.82 (1.75–1.92)	1.66 (1.60–1.76)	1.76 (1.63–1.88)		1.79 (1.65–1.88)	1.65 (1.63–1.68)
Mass (kg)		66 (56–90)	78 (66–90)	58 (56–64)	79 (51–127)		79 (73–127)	76 (51–100)
BMI (kg/m^2^)		22.1 (18.3–29.4)	23.6 (18.3–29.4)	21.5 (18.4–23.5)	27.3 (19.2–37.5)		27.3 (20.7–37.5)	27.4 (19.2–35.6)
*Max Calf Circumference* (mm)								
Residual		–	–	–	290 (250–450)		300 (260–450)	270 (250–290)
Contralateral		360 (320–410)	390 (350–410)	360 (320–360)	390 (340–530)		390 (350–530)	390 (340–440)
Residual limb length (mm)		–	–	–	150 (100–300)		150 (100–300)	210 (140–270)
Time since amputation (years)		–	–	–	7.5 (1–35)		5.0 (1–35)	18.5 (8–29)
*Amputation cause*								
CRPD		–	–	–	2		1	1
Congenital		–	–	–	2		1	1
Trauma		–	–	–	5		5	0
PVD		–	–	–	1		1	0
Daily socket use (h)		–	–	–	12.5 (6–16)		11.5 (6–616)	14.5 (14-15)

### Soft Tissue Composition

**Figure 3:**
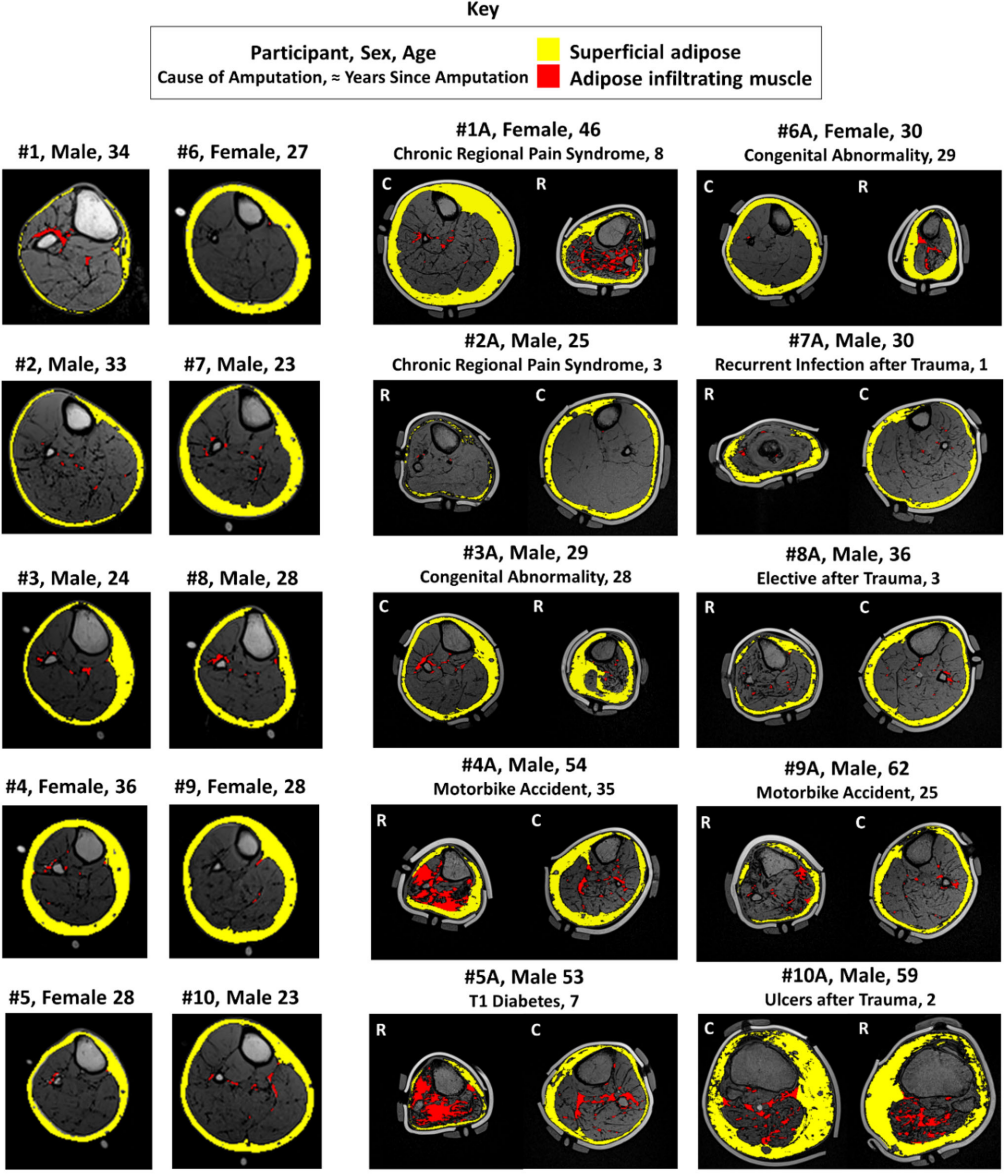
Exemplar transverse MRI slices in the calf with superficial adipose (yellow) and adipose infiltrating muscle (red) tissue overlays. Images represent the right control limb of ten participants without amputation (left columns #1-10), and both the control (C) and residual (R) limbs for ten participants with transtibial amputation (right columns, #1A-#10A).

**Figure 4:**
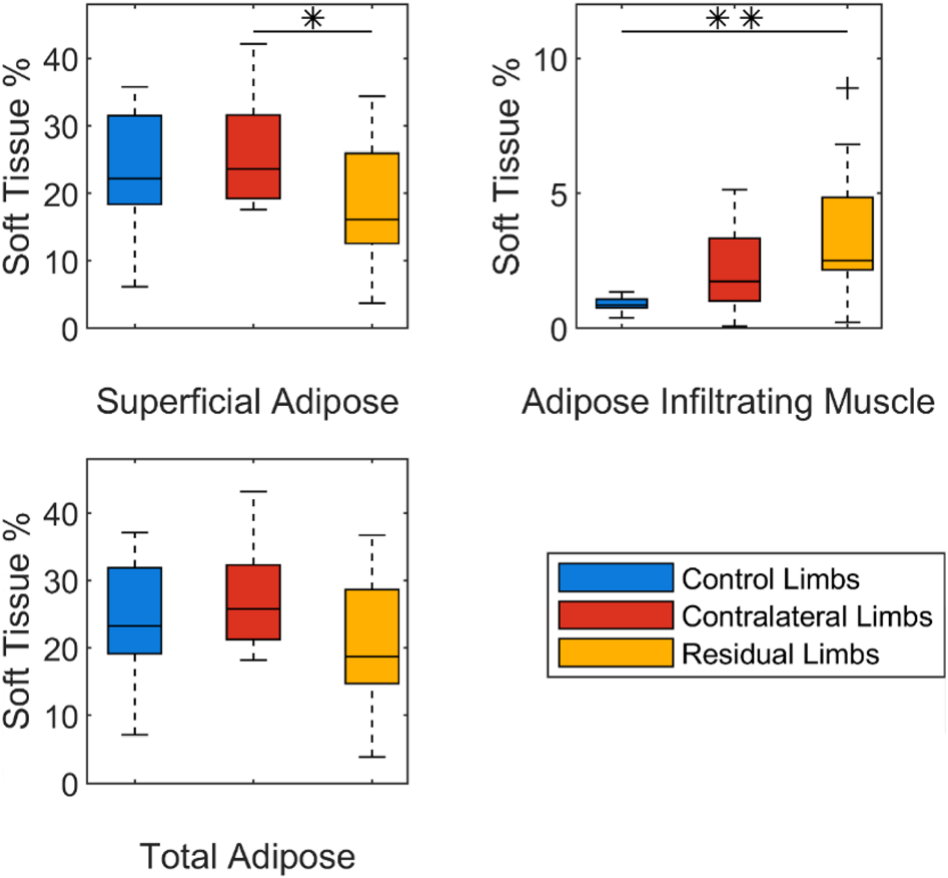
Median, interquartile range (IQR) and range in percentage of tissue constituents of the overall limb, in a 60mm segment distal from the tibial plateau. + indicates outliers; * indicates significance at *p* ≤ 0.05; ** indicates significance at *p* ≤ 0.01.

Residual limbs were observed to have a smaller cross-sectional area and a less consistently round shape than intact limbs, although the residual limbs often revealed distorted shape artefacts resulting from the foam support (Fig. 3). Figures in the Supplementary Data detail the percentage volumes of superficial adipose tissue, adipose infiltrating muscle and muscle tissue across the limb sections for all participants. Residual limbs displayed greater adipose tissue infiltrating muscle than intact limbs (Fig. 3), reaching significance (*p* < 0.05) when compared to the residual and control limbs (Fig. 4).

**Figure 5:**
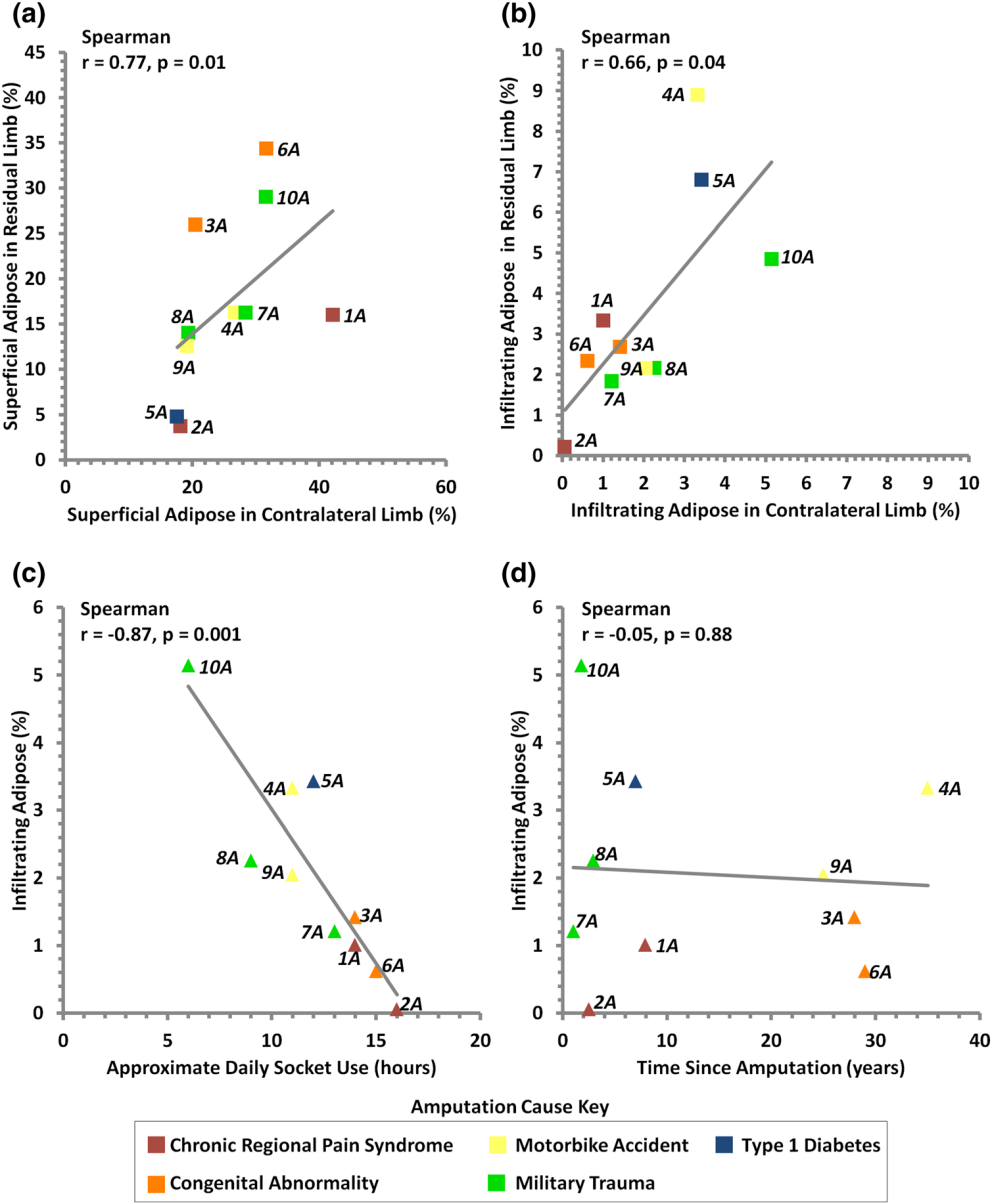
Positive correlations were observed between residual limb and contralateral limb superficial adipose (a) and infiltrating adipose (b). Negative correlation was observed between percentage volume of infiltrating adipose tissue in contralateral limbs and estimated daily socket use (c), though no correlation was seen between contralateral limb infiltrating adipose and time since amputation (d). Number indicates participant ID.

Correlation analysis was performed between the percentage volumes of adipose tissue, and three intrinsic factors, namely BMI, time since amputation and daily socket use (Fig. 5, Table 2). A significant positive correlation was observed between the levels of both adipose tissue types in the residual and contralateral limbs (Fig. 5a and b). There was a negative correlation between adipose infiltrating muscle and estimated daily socket use in both contralateral and residual limbs, although this was only statistically significant for the former (*r * =  − 0.87, *p* < 0.01, Fig. 5c). In contrast, no correlation was evident between the adipose infiltrating muscle values and the time since amputation (*r*  =  − 0.05, *p*  =  0.88, Fig. 5d). It was also interesting to note that there was a positive trend between the adipose infiltrating muscle tissue and the BMI in the control, non-amputated group, although the correlation was not statistically significant (*r*  =  0.46, *p* =  0.18). With respect to superficial adipose values, there were no significant correlations with any of the three intrinsic factors (Table 2).

**Table 2:** Correlation analysis for selected intrinsic factors (BMI, time since amputation and estimated daily socket use) and the percentage volume of infiltrating and superficial adipose from the tibial plateau to 60mm distally, in the right control limbs of ten participants without amputation and the contralateral and residual limbs of ten participants with unilateral transtibial amputation. Bold text and ** represents significance at the 1% level.

Correlation between		Limb	Correlation *r*	Significance *p*
Percentage volume of infiltrating adipose and:	BMI	Control	0.46	0.18
		Contralateral	0.35	0.33
		Residual	0.12	0.75
	Time Since Amputation	Contralateral	− 0.05	0.88
		Residual	0.45	0.19
	Est. Daily Socket Use	Contralateral	− 0.87	<0.001*
		Residual	− 0.34	0.34
Percentage volume of superficial adipose and:	BMI	Control	0.12	0.75
		Contralateral	0.08	0.83
		Residual	− 0.19	0.60
	Time Since Amputation	Contralateral	0.12	0.75
		Residual	0.20	0.59
	Est. Daily Socket Use	Contralateral	0.09	0.82
		Residual	− 0.04	0.92

### Interface Pressure

At a cuff inflation pressure of 60 mmHg, the mean interface pressures ranged from 66 to 74 mmHg, 70 to 75 mmHg and 72 to 84 mmHg in control, residual and contralateral limbs, respectively (Table 3). The highest pressures and variability generally occurred at the patellar tendon, which represented the measurement site with the lowest soft tissue coverage over the underlying bony anatomy.

**Table 3:** Interface pressure at three measurement sites, at baseline and a cuff pressure of 60mmHg, applied to the right control limb of 10 participants without amputation and both residual and contralateral limbs of 10 participants with unilateral transtibial amputation

	Measurement site	Mean (S.D.) interface pressure at applied cuff pressure	
		0 mmHg (Baseline)	60 mmHg
Control limbs	Patella Tendon	13.1 (7.4)	73.7 (8.2)
	Lateral Calf	4.7 (2.9)	72.6 (5.5)
	Posterior Calf	0.5 (1.3)	66.2 (5.0)
Contralateral limbs	Patella Tendon	17.7 (15.2)	83.6 (34.3)
	Lateral Calf	7.7 (11.1)	75.1 (6.7)
	Posterior Calf	2.8 (6.3)	72.0 (11.7)
Residual limbs	Patella Tendon	13.6 (13.4)	73.1 (21.6)
	Lateral Calf	13.9 (12.4)	75.1 (11.4)
	Posterior Calf	11.9 (13.4)	69.9 (12.7)

### Soft Tissue Deformation and Strain

**Figure 6:**
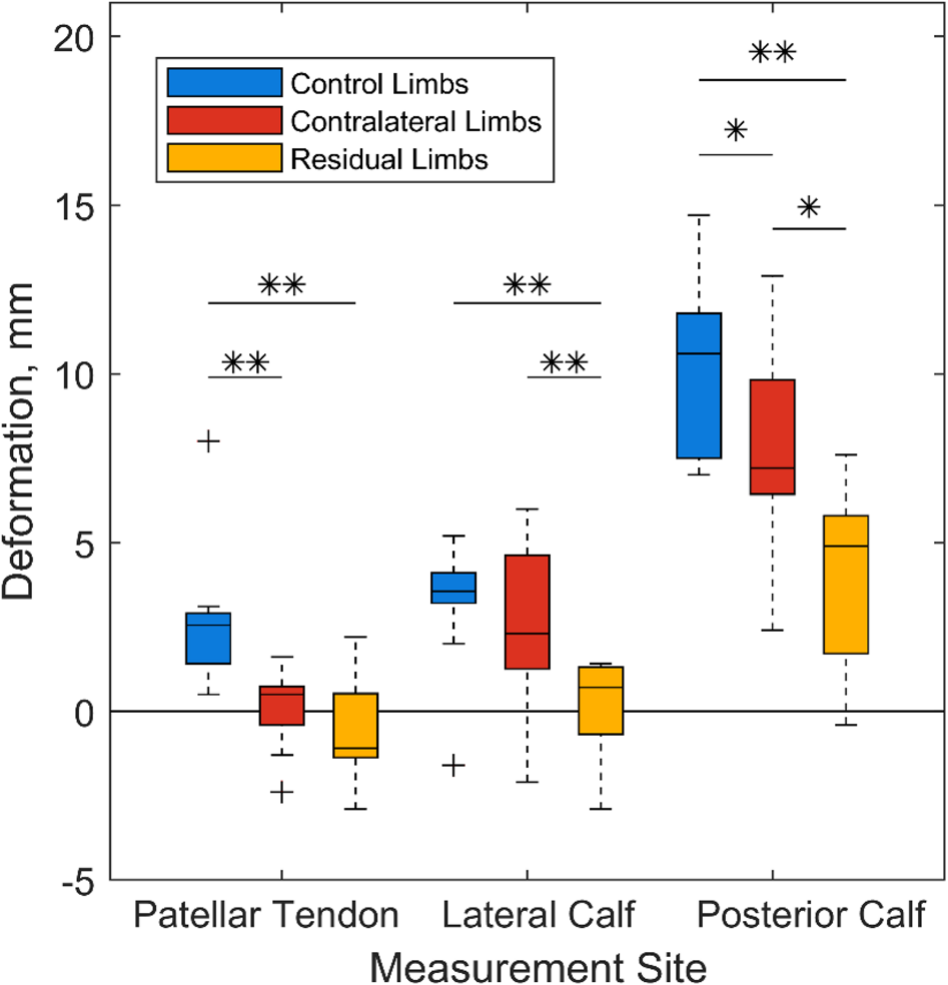
Median, interquartile range (IQR) and range of lower limb soft tissue deformation under 60mmHg pressure cuff loading for all participant groups. * indicates significance at *p* ≤ 0.05 and ** indicates significance at *p* ≤ 0.01.

The soft tissue shape changes from baseline to a cuff pressure of 60 mmHg (8 kPa), visualised from MR images (Figures in the Supplementary Data) were converted into two parameters, gross tissue deformation and strain. These data revealed that deformation was significantly higher (*p* < 0.01) in control limbs than residual limbs at all three sites (Fig. 6). Deformation was also higher in control limbs than the contralateral limbs, with statistically significant differences at the patellar tendon (*p* < 0.01) and the posterior calf (*p* < 0.05). Within the individuals with amputation, deformation was significantly different between their residual and contralateral limbs at the lateral (*p* < 0.01) and posterior calf sites (*p* < 0.05). Strain results revealed similar trends to that of deformation. However, there were no significant differences between groups at the posterior calf site, with all three sites observed to demonstrate similar strain magnitudes. The high deformation at the posterior calf site produced relatively low strains owing to its high soft tissue layer thickness. Few notable correlations were revealed between either gross tissue deformation or compressive strain and percentage volume of superficial adipose or time since amputation (Supplementary Data Table S1).

### Soft Tissue Stiffness

**Figure 7:**
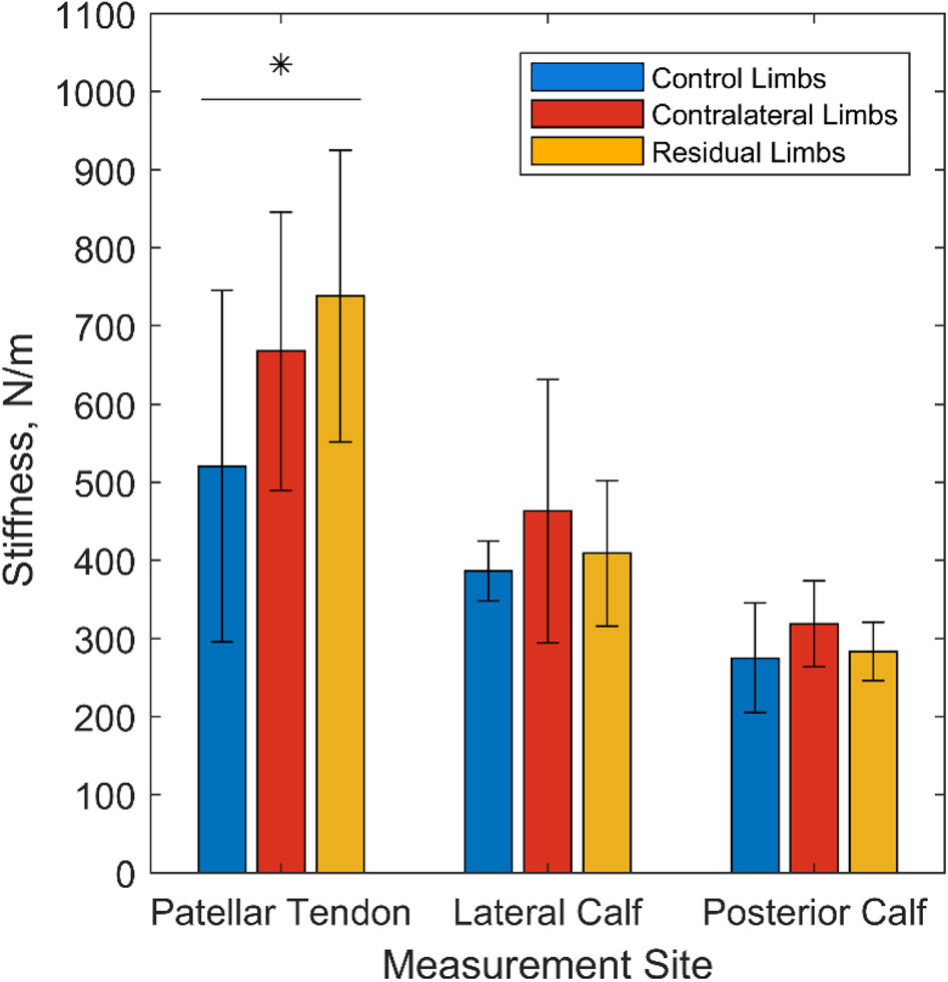
Mean values of tissue structural stiffness at three measurement sites on the right control limb of eight participants without amputation and both contralateral and residual limbs of ten participants with unilateral transtibial amputation. * indicates *p* ≤ 0.05.

Structural stiffness values were highest at the patellar tendon site, which has the least soft tissue coverage, adjacent to a bony prominence (Fig. 7). The highest stiffness values (mean 740 ± 190 N/m) were observed in the residual limb group at the patella site, which were significantly higher than those estimated from the control group (*p* < 0.05). No differences were observed between groups in the lateral calf or posterior calf, and few notable correlations were revealed between structural stiffness and percentage volume of superficial adipose or time since amputation (Supplementary Data Table S2).

## Discussion

This study was designed to investigate residual limb soft tissue composition and how loading affects tissue deformation. It presents, for the first time, the combination of MRI data and structural stiffness measurements using a commercial device. Two cohorts were recruited, with and without transtibial amputation, and they were imaged using MRI prior to and during the application of representative mechanical loads via a pressure cuff. The results revealed significant changes to soft tissue composition in the residual limb, with a higher proportion of muscle-infiltrating adipose tissue, which was associated with the amount of daily socket use. A critical load bearing site, the patellar tendon, was also shown to demonstrate significantly increased stiffness in the residual limb compared to intact control limbs.

### Measurements and Analysis

MRI data enabled clear visualisation of the soft tissues with the specific distinction of bone, muscle and adipose tissues (Figs. 3, 4). Comparison between the control and amputee groups demonstrates how pathology resulted in a markedly increased variability in limb tissue composition and morphology. Residual limbs contained approximately three times more infiltrating adipose tissue than control limbs of participants without amputation. Adipose infiltrating muscle was particularly apparent in more established residual limbs (#4A, #6A and #9A), and in two people with shorter time since amputation (#1A and #5A). One of these participants (#1A) used a wheelchair for mobility for several years prior to amputation which may have caused additional atrophy in the lower limb muscles, and the other (#5A) had Type 1 diabetes which has been associated with increased adipose infiltrating muscle.^5^ These observations reflect the well-established changes in tissue composition associated with muscle atrophy post-amputation associated with denervation and disuse.^28,51,58^ However, this study has proved novel in discriminating between superficial and adipose infiltrating muscle tissues in residual limbs, thus providing insight into the potential for both disease progression^1,20,21^ and an enhanced risk for DTI.^37,52,57^

Correlation analysis provided insights into the relationships between tissue composition in the contralateral and residual limbs and intrinsic factors associated with the individuals post-amputation (Table 2). Residual limb adipose was observed to correlate significantly with contralateral limb adipose for both superficial and infiltrating types (Fig. 5 top). However, a high percentage volume of superficial adipose tissue did not necessarily correspond with high infiltrating adipose indicating that various factors may be responsible for the infiltration. It is of note that superficial adipose did not correlate with BMI, time since amputation or estimated hours of prosthesis use. By contrast, a significant negative correlation was revealed between infiltrating adipose in contralateral limbs and estimated daily prosthesis usage, which supports the suggestion that infiltrating adipose may represent a biomechanical adaptation, namely muscular atrophy due to disuse. More active limbs presented with more lean muscle mass, and the lack of correlation for residual limbs may indicate the influence of other factors such as gait compensations where participants favour their intact limb.^30^ Adipose tissues change in size and function in response to a number of factors including loading, exercise, temperature and nutrition, with hypertrophy observed under static tension.^27,61^ This may have influenced the structural stiffness values measured and is worthy of further exploration.

The composition and status of soft tissues will affect how they respond to and tolerate mechanical loading. The pressure cuff was used to apply pressure representative of PPAM aid use during rehabilitation.^47^ Loading was applied through a 60 mmHg cuff inflation, with some non-uniformity of interface pressures. The patellar tendon, with its relatively thin soft tissue coverage, demonstrated the highest pressures. Low, non-zero pressure was measured between the indenters and limb at baseline (0mmHg cuff pressure) although these were within the reported errors of the pressure measurement system.^4^ Using MRI to evaluate tissue deformation pre- and post-loading, and resulting gross compressive strain, revealed the lowest values at the residual limb patellar tendon site. This corresponded to the highest tissue stiffness measurements (Fig. 7), and is the location at which many prosthetists focus loading (i.e. using patella tendon bearing socket designs^44^), and could be attributed to local biomechanical adaptation in response to repetitive loading at this location. Residual limbs were also generally smaller than contralateral limbs, resulting in higher compressive strains for equivalent deformations at residual limb sites (Fig. 6). At the calf sites, the highest stiffness values were observed in the contralateral limbs, which could again indicate adaptation in response to compensatory gait patterns, prior to- or following amputation.^30^ Though the MyotonPRO assesses the superficial tissues only, it produced structural stiffness results consistent with gross mechanical indentation in the transtibial amputated limb’s anterior aspect (approximately 400–500 N/m).^49^ The recorded structural stiffness values were in the same range as reported in other myotonometry studies of skeletal muscles in the lower limb. These studies reported mean values ranging from approximately 275–450 N/m for the gastrocnemius,^13,22,24,26^ and 350–400 N/m for the tibialis anterior,^24,26^ which correspond with the present study’s posterior calf and lateral calf sites, respectively. Literature studies reporting MyotonPro assessment of the patella tendon have primarily recruited athletes, for whom the stiffness values might be elevated. However, neglecting elite athlete studies and considering control groups, mean values have been reported ranging from 780 to 900 N/m,^7,60^ consistent with the present study’s results.

During periods of loading application, the magnitude and duration of mechanical strain is considered to represent the most important factor in the causal pathway for damage of soft tissue.^8,15,31–33,39,40,53,54^ The largest strains observed in this study were between 20 and 30%, applied over a 15 minute period. These conditions represent a lower range than that observed in examining tissue damage in model systems^17^ and in clinical situations.

### Limitations

The study’s generalisability is limited by its small sample size and heterogeneity of the individuals with amputation. The group has a substantial range in time since amputation (1–35 years), although this variety is largely representative of the community accessing prosthetics services in our Southern UK area. Accepting this limitation, the study was designed to evaluate the heterogeneous nature of a cohort with lower limb amputations with a wide range of individual demographics, reasons for amputation and associated tissue morphologies and responses. Accordingly, the study used correlation analysis and prioritised comparison between the residual and contralateral limbs. Thus, the present study has provided insight into the factors that can affect tissue adaptation and load tolerance post-amputation, with further studies needed to explore how these differences could affect their tolerance to prosthetic loading. The two cohorts were not matched by their demographics (age, weight and BMI in particular) and this may substantially affect the comparisons observed between the people with amputations and the intact controls. However, the intact control data are interesting to assess the soft tissue characteristics of a young, healthy group of people without amputations, whose lower limb soft tissues might represent the start point of prosthetic rehabilitation in cases of amputation due to trauma or neoplasia. The sample size is a recurring issue for lower limb prosthetics studies with a small population of eligible participants, so the presented data do not cover the full variance of physiological conditions experienced in the wider population of people who use prosthetics. The present findings provide focus for further study on more homogeneous groups of particular interest or concern.

During the testing sessions it was often difficult to support residual limbs in a consistent manner, and participants with short residual limbs and knee flexion contracture required support from below. These factors dictated the length of all the limbs which could be consistently imaged, at ≈ 60 mm. With respect to structural measurements, although limbs were kept in a consistently supported position, relaxation of the muscles was not achieved objectively. Indeed, contracted muscles could have presented with higher stiffness and elasticity values.^14^ Furthermore, the MyotonPRO measurement system is mainly designed for measuring the stiffness of superficial tissues, so any adaptation of deeper muscular tissues may be less apparent in participants with higher superficial adipose tissue.^9^ Considering tissue strain measurement, the reported values are equivalent to a measure of engineering strain. Therefore, these estimates are not directly comparable to the principal and shear Green-Lagrange strain components most commonly employed in imaging and finite element analysis (FEA) estimation of tissue damage risk thresholds.^8,31,32,54^ The paired, aligned MR images collected in this study would enable Green-Lagrange strain prediction using FEA but this was outside the present scope.

### Summary and Clinical Implications

A higher proportion of muscle-infiltrating adipose was observed in residual limbs compared to intact limbs, indicating muscle atrophy post-amputation. Residual limbs were also stiffer at the patellar tendon site and demonstrated less strain under external pressure than intact limbs. Understanding the changes in tissue composition can provide clinicians with new insights into how residual limb tissues adapt to representative prosthetic loading and could offer strategies to prevent skin and sub-dermal damage, which is common in this population.

The evidence of superficial tissue biomechanical adaptation in response to increased mechanical loads, notably at the patellar tendon, extends evidence from case studies reporting increased soft tissue tolerance to ischemia under loading at this critical location which is exploited by prosthetists for residuum-prosthesis load transfer.^6^ The results also show indicators of muscle atrophy, presenting as elevated adipose infiltrating muscle tissue in residual limbs. This is a well-established marker of pressure ulcer risk in individuals with spinal cord injury, and accumulates over time,^16^ so this evidence contributes to our understanding of these individuals’ risk of deep tissue injury, as well as metabolic syndrome, cardiovascular disease and related mortality.^38^

This study demonstrates how residual limb soft tissues can change post-amputation in a small population with a range of amputation causes, and longitudinal studies could help to determine more predictive variables that affect tissue composition and tolerance to loading post-amputation. This insight will help to further understanding of how the soft tissues adapt to tolerate prosthetic loading, to help reduce the risk of tissue damage during prosthetic use.
